# Genetic and clinical findings of panel‐based targeted exome sequencing in a northeast Chinese cohort with retinitis pigmentosa

**DOI:** 10.1002/mgg3.1184

**Published:** 2020-02-26

**Authors:** Yan Sun, Wei Li, Jian‐kang Li, Zhuo‐shi Wang, Jin‐yue Bai, Ling Xu, Bo Xing, Wen Yang, Zi‐wei Wang, Lu‐sheng Wang, Wei He, Fang Chen

**Affiliations:** ^1^ Shenyang He Eye Specialist Hospital Shenyang China; ^2^ He University Shenyang China; ^3^ BGI Education Center University of Chinese Academy of Sciences Shenzhen China; ^4^ BGI‐Shenzhen Shenzhen China; ^5^ Department of Computer Science City University of Hong Kong Kowloon Hong Kong; ^6^ Guangdong Provincial Key Laboratory of Human Disease Genomics Shenzhen Key Laboratory of Genomics BGI-Shenzhen Shenzhen China; ^7^ School of Basic Medicine Qingdao University Qingdao China

**Keywords:** compound heterozygous, digenic inheritance, panel‐based targeted exome sequencing, retinitis pigmentosa

## Abstract

**Background:**

Panel‐based targeted exome sequencing was used to analyze the genetic and clinical findings of targeted genes in a cohort of northeast Chinese with retinitis pigmentosa.

**Methods:**

A total of 87 subjects, comprising 23 probands and their family members (total patients: 32) with confirmed retinitis pigmentosa were recruited in the study. Panel‐based targeted exome sequencing was used to sequence the patients and family members, all subjects with retinitis pigmentosa underwent a complete ophthalmologic examination.

**Results:**

Of the 23 probands, the clinical manifestations include night blindness, narrowing of vision, secondary cataracts, choroidal atrophy, color blindness, and high myopia, the average age of onset of night blindness is 12.9 ± 14 (range, 0–65; median, 8). Posterior subcapsular opacities is the most common forms of secondary cataracts (nine cases, 39.1%), and peripheral choroidal atrophy is the most common form of secondary choroidal atrophy (12 cases, 52.2%). Of these probands with complication peripheral choroidal atrophy, there were eight probands (66.7%, 8/12) caused by the pathogenic variation in *USH2A* gene. A total of 17 genes and 45 variants were detected in 23 probands. Among these genes, the commonest genes were *USH2A* (40%; 18/45), *RP1* (15.6%; 7/45), and *EYS* (8.9%; 4/45), and the top three genes account for 56.5% (13/23) of diagnostic probands. Among these variants, comprising 22 (48.9%) pathogenic variants, 14 (31%) likely pathogenic variants, and nine (20%) uncertain clinical significance variants, and 22 variants was discovered first time. Most of the mutations associated with RP were missense (53.3%, 24/45), and the remaining mutation types include frameshift (35.6%, 16/45), nonsense (6.7%, 3/45), and spliceSite (4.4%, 2/45). Among the probands with mutations detected, compound heterozygous forms was detected in 13 (56.5%, 13/23) probands, and digenic inheritance (DI) forms was detected in five (21.7%, 5/23) probands.

**Conclusion:**

Panel‐based targeted exome sequencing revealed 23 novel mutations, recognized different combinations forms of variants, and extended the mutational spectrum of retinitis pigmentosa and depicted common variants in northeast China.

## Graphical abstract

We applied panel‐based targeted exome sequencing to explore the pathogenic variation spectrum and genetic characteristics of retinitis pigmentosa populations in northeast China, and have a deeper understanding of the relationship between clinical manifestations and genotypes.

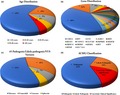

## 1 INTRODUCTION

Retinitis pigmentosa (RP, MIM#500004) is the most common group of disorder in retinal diseases, with a common manifestation of progressive photoreceptor cells and loss of retinal pigment epithelial function. Retinitis pigmentosa has a strong clinical heterogeneity (Ayuso & Millan, 2010; Galan et al., 2011). The early typical features mainly start from the peripheral retina and gradually develop into the fovea. The main symptoms include night blindness, progressive reduction of visual field, and ultimately tubular vision and blindness. It is the most common blinding monogenic hereditary fundus disease with a prevalence of approximately 1/3,500–1/5,000 (Galan et al., 2011). The pathogenesis of retinitis pigmentosa is closely related to genetic factors. Different gene mutations have different clinical phenotypes, and the specific pathogenesis research is still unclear (Schuster, 2008).

The clinical and genetic heterogeneity of retinitis pigmentosa is difficult for clinical diagnosis and differential diagnosis. Panel‐based targeted exome sequencing has been widely used in genetic disease screening and clinical diagnosis. With the continuous advancement of this technology and the continuous reduction in the cost of genetic testing, genetic detection‐assisted diagnosis of patients with retinitis pigmentosa and family members has become a reality. Despite the development of targeted sequencing screening strategies for identifying known genes associated with RP, it is estimated that 40% of cases are still not molecularly diagnosed, indicating that there are still many novel mutations in known disease‐causing genes and novel retinitis pigmentosa diseases‐causative gene not found (Ellingford et al., 2016). Here, we reported the mutational spectrum of 23 probands diagnosed with retinitis pigmentosa. The characteristics and clinical manifestations of retinitis pigmentosa in the northeast Chinese population were analyzed, which provided assistance for subsequent clinical diagnosis and genetic counseling.

## 2 MATERIALS AND METHODS

### 2.1 Subjects and ethics statement

A total of 87 subjects from 23 families with retinitis pigmentosa diagnosed in Shenyang He Eye Specialist Hospital from January 2017 to July 2018 were recruited in the study. Among these subjects, including 44 males and 43 females, aged 9–88 years old, with a median of 36 years old. Inclusion criteria for patients: (a) night blindness; (b) gradual decline in visual acuity; (c) typical fundus changes, optic disc with waxy yellow atrophy, retinal osteoblast‐like pigmentation, thinning of blood vessels, blue–gray retina; (d) The early peripheral visual field exhibits a circular dark spot, and the late visual field narrows toward the center; (e) After dark adaptation and light adaptation in the early stage of the lesion, the full‐field electroretinogram (ERG) examination showed a decrease in rod function, and decrease in cone function at the same time in the late stage (Hartong, Berson, and Dryja, 2006; Xu, Hu, Ma, Li, and Jonas, 2006). All the examinations and tests involved in this study were approved by the Ethics Committee of Shenyang He Eye Specialist Hospital (approval number: IRB (2016) K001.01), following the Helsinki Declaration, and obtaining informed consent from patients and family members.

### 2.2 Clinical assessment

Both patients and family members underwent a comprehensive clinical examination to confirm the diagnosis and to exclude ocular diseases caused by nongenetic factors. Clinical examinations include medical history inquiries and physical examinations. Among them, medical history inquiry includes basic personal information (including gender, age, place of origin, ethnicity, etc.), chief complaint, current medical history (age of onset, regularity, treatment status, medication status, type and time of complications, etc.), past history (full body Situation, history of genetic testing, etc.), family history, history of surgery and drug use, history of marriage and childbirth. Physical examination includes vision and corrected visual acuity, non‐contact intraocular pressure, color vision, B‐ultrasound (Tianjin Mida, model ODM‐2200), visual field (Carl Zeiss, model 750i), anterior segment photography, fundus color photography (Topcon, model TRC NW‐300), optical coherence tomography (OCT) (macular, optic nerve layer thickness, Angiography) (Cirrus HD‐OCT 5000), and full‐field electroretinography (ERG). In addition, patients also underwent conventional elbow venous blood collection.

### 2.3 Panel‐based targeted exome sequencing

All eligible patients and family members routinely collected 5 ml of elbow venous blood, ETDA anticoagulation, and stored at −80°C. The DNA was extracted from whole blood using the FlexiGene DNA Kit (Qiagen) according to the manufacturer's protocols. We designed a Panel‐based high‐throughput targeted enrichment method to capture exon capture regions of 792 genes associated with common Inherited eye diseases (Table S1). The Capture Panel (Target_Eye_792_V2 chip) was custom designed and produced by the Beijing Genomics Institute (BGI) (Gao, Li, et al., 2019; Gao, Qi, Hu, Wang, & Wu, 2019; Hu et al., 2019; Li et al., 2019). To obtain the probe sequence, we obtained the exon sequence of 792 genes and its flanking ±30 bp from the reference human genome (UCSC hg 38). On average, the mean coverage depth was more than 300X, and the coverage of target region was ~99.9% using BGISEQ‐2000 (BGI, Inc.).

### 2.4 Bioinformatics analysis

We aligned sequence reads to the reference human genome (UCSC hg38) using the Burrows–Wheeler aligner version 0.7.10 (BWA‐MEM). Analysis of the data obtained using previous similar research methods (Gao, Li, et al., 2019). Using the Human Gene Mutation Database, Online Mendelian Inheritance in Man, and the ClinVar database and reference literature reports identified previously reported variations. According to the standards of the American College of Medical Genetics (ACMG), Variants was classified as pathogenic, likely pathogenic, and novel variants of uncertain clinical significance (Richards et al., 2015). Four online function prediction software was used to assess the potential deleterious of the variation, including SIFT (SIFT, http://sift.jcvi.org/), MutationTaster (http://www.mutationtaster.org/), FATHMM (http://fathmm.biocompute.org.uk/), and LRT (http://www.genetics.wustl.edu/jflab/). The obtained candidate variants were first verified by Sanger sequencing or quantitative real time polymerase chain reaction, then reviewed by clinical geneticists and ophthalmologists, and validation of variant segregates with the disease within the family.

## 3 RESULTS

### 3.1 Clinical presentation and genetic finding

**Figure 1:**
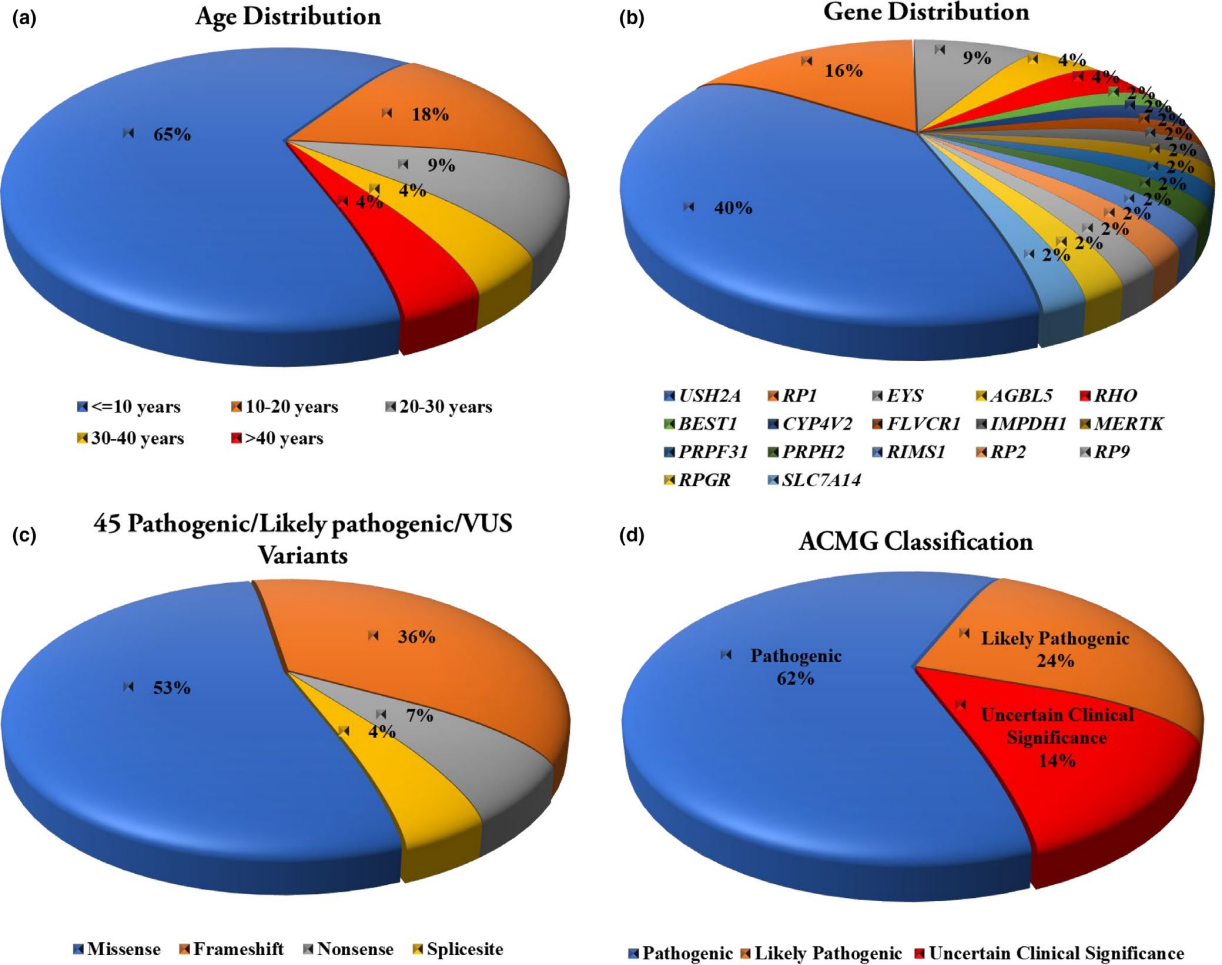
Basic information of clinical presentation and genetic finding of the target exome sequencing in the patients. (a) The age distribution of the total Patients, including age ≤10 years (*n* = 15), 10–20 years (*n* = 4), 20–30 years (*n* = 2), 30–40 years (*n* = 1), and >40 years (*n* = 1); (b), Distribution of retinitis pigmentosa (RP)‐causative genes in the 32 patients. Mutations were identified in 17 genes, with 65% of the mutations found in the top three genes (USH2A, RP1, EYS). (c) Forty‐five pathogenic/likely pathogenic/uncertain clinical significance variants were identified, including missense (*n* = 24), frameshift (*n* = 16), nonsense (*n* = 3), and splicing (*n* = 2) variants. (d) Forty‐five pathogenic/likely pathogenic/uncertain clinical significance variants were identified, including pathogenic (*n* = 22), likely pathogenic (*n* = 14), and uncertain clinical significance (*n* = 9) variants. Of these variants, 23 of them were described for the first time

In this study, there were 23 families (23 probands and their relatives), comprising 32 patients with clinically diagnosed retinitis pigmentosa and 55 subjects with normal visual acuity. Among the 32 patients, six of them had best corrected visual acuity of both eyes greater than 0.1, nine of them had the best corrected visual acuity of both eyes less than finger count (FC), and the remaining patients were between the two level (FC ~0.1). Among the 23 probands, the average age of probands was 48.2 ± 17.7 (range, 21–81; median, 43), and the average age of onset of night blindness was 12.9 ± 14 (range, 0–65; median, 8), of which the majority age of onset of night blindness were under 10 years old, accounts for 65% (15/23) (Figure 1a). The average duration of disease in the 23 probands was 35.3 ± 17.3 (range, 13–72; median, 31). Clinical manifestations of different forms of concurrent lens opacities was found in 17th probands, including nine probands with posterior subcapsular opacities, four probands with anterior subcapsular opacities, two probands with punctate opacities, and two probands with nuclear opacities. Clinical manifestations of different forms concurrent choroidal atrophy was found in 21 probands, including 12 probands with peripheral choroidal atrophy, five probands with total choroidal atrophy, and four probands with posterior choroidal atrophy. Of these probands with peripheral choroidal atrophy, the results showed that eight (66.7%, 8/12) of these probands were caused by the mutation of the *USH2A* gene. A total of 13 probands with systemic or other ocular manifestations, including five probands with color blindness and two probands with high myopia. Clinical information of the probands with definitive diagnosis is shown in Table 1.

**Table 1:** Basic information of clinical presentation in 23 probands with retinitis pigmentosa

Family ID	Sex	Age at examination (Yr)	BCVA (OD, OS)	Clinical diagnosis	History	Age of onset (Yr)	Lens opacity	Choroidal atrophy	Other ocular manifestation
1	M	23	FC/FC	Retinitis pigmentosa	N	6	N	N	Retinal detachment
2	F	55	0.02/0.3	Retinitis pigmentosa	N	4	Posterior subcapsular	Peripheral	N
3	M	81	0.05/FC	Retinitis pigmentosa	N	65	Nuclear	Total choroid	N
4	F	62	0.01/0.03	Retinitis pigmentosa	Y	5	Nuclear	Peripheral	Red–green color blindness
5	F	79	0.1/HM	Retinitis pigmentosa	Y	7	Anterior subcapsular	Posterior pole	blue color blindness
6	M	39	HM/HM	Retinitis pigmentosa	Y	5	Posterior subcapsular	Posterior pole	N
7	F	37	0.6/0.8	Retinitis pigmentosa	N	12	N	N	Retinal crystal deposit
8	F	68	HM/HM	Retinitis pigmentosa	N	15	Posterior subcapsular	Posterior pole	N
9	F	33	0.6/0.5	Retinitis pigmentosa	N	12	N	Peripheral	Hypotension
10	M	45	0.1/0.05	Retinitis pigmentosa	N	8	Posterior subcapsular	Peripheral	N
11	F	56	FC/HM	Retinitis pigmentosa	N	30	Posterior subcapsular	Peripheral	Hearing loss
12	M	72	HM/HM	Retinitis pigmentosa	N	7	Anterior subcapsular	Posterior pole	N
13	F	39	HM/HM	Retinitis pigmentosa	N	8	punctate	Total choroid	Macular hole
14	M	21	HM/HM	Retinitis pigmentosa	N	0	Posterior subcapsular	Total choroid	Total color blindness
15	M	29	HM/HM	Retinitis pigmentosa	N	9	Posterior subcapsular	Total choroid	High myopia
16	F	66	0.3/0.25	Retinitis pigmentosa	Y	8	Posterior subcapsular	Peripheral	N
17	F	38	0.15/0.5	Retinitis pigmentosa	N	25	N	Peripheral	N
18	M	64	0.05/0.05	Retinitis pigmentosa	Y	32	Posterior subcapsular	Peripheral	Ménière disease
19	M	43	0.1/0.2	Retinitis pigmentosa	Y	8	N	Peripheral	N
20	F	31	0.1/0.2	Retinitis pigmentosa	N	14	punctate	Peripheral	blue color blindness
21	M	36	0.05/0.05	Retinitis pigmentosa	N	5	N	Peripheral	Red–green color blindness
22	M	36	0.05/0.1	Retinitis pigmentosa	N	8	Anterior subcapsular	Peripheral	N
23	M	56	HM/HM	Retinitis pigmentosa	Y	3	Anterior subcapsular	Total choroid	High myopia

Abbreviations: F, female; FC, finger count; HM, high myopia; M, male; N, No; Y, Yes.

A total of 45 pathogenic/likely pathogenic/uncertain clinical significance variants were identified among 17 retinitis pigmentosa (RP)‐causative genes, including pathogenic (*n* = 22), likely pathogenic (*n* = 14), and uncertain clinical significance (*n* = 9) variants, with 65% of the mutations found in the top three genes (*USH2A*, MIM#608400; *RP1*, MIM#603937; *EYS, MIM*#612424), and the top three genes account for 56.5% of diagnostic probands.. Among these variants, including missense (*n* = 24), frameshift (*n* = 16), nonsense (*n* = 3), and splicing (*n* = 2) mutations, 23 of them were described for the first time (Figure 1b–d). Among the probands with mutations detected, compound heterozygous forms was detected in 13 (56.5%, 13/23) probands, and digenic inheritance forms was detected in five (21.7%, 5/23) probands. Of them, the most common compound heterozygous forms is the mutation of different alleles of the *USH2A* gene (61.5%, 8/13). Consistent with previous studies, most RP patients carried compound heterozygous variants, but few patients detected digenic gene variants. In addition, five variants of three genes were detected in one proband, and four variants of the *RP1* gene were detected in another one proband. Among the first discovered candidate pathogenic mutations, seven unreported mutations were found in the *USH2A* gene, and five unreported mutations were found in the *RP1* gene. Two unreported candidate pathogenic mutations were found for the *AGBL5* gene and the *EYS* gene, respectively. A novel candidate pathogenic variation was detected in the *FLVCR1* (MIM#609144), *MERTK* (MIM**#**604705), *PRPF31* (MIM#606419), *RP2* (MIM#300757), *RP9* (MIM#607331), and *RPGR* (MIM#312610) genes, respectively (Table 2).

**Table 2:** Genetics finding in the probands with retinitis pigmentosa

Family ID	Gene	MutName	Amino acid change	Exon Intron ID	Zygous	Chr:por:mut	Functional change	SIFT	Polyphen2	Mutation taster	Clinical significance	Inheritance mode	References
1	*IMPDH1*	c.942_944delGAA	p.Lys314del	EX10/CDS10	Het	chr7:128398543:GTTC > G	Frameshift	.	.	.	P	AD	Jin, Qu, Qu, Meng, Xu, & Yin, 2014
2	*SLC7A14*	c.1391G > T	p.Cys464Phe	EX7/CDS6	Het	chr3:170198680	Missense	Dam	Neu	.	P	AR	Jin, Huang, et al., 2014
	*PRPF31*	c.357_358delAA	p.Ser119SerfsX5	EX5/CDS4	Het	chr19:54625910.0.54625911	Frameshift	.	.	.	P	AR	Jianping et al., 2018
3	*PRPH2*	c.205delG	p.Val69CysfsX30	EX1/CDS1	Het	chr6:42689868	Frameshift	.	.	.	P	AD	Manes et al., 2015
4	*RHO*	c.403C > T	p.Arg135Trp	EX2/CDS2	Het	chr3:129530917:C > T	Missense	.	DC	PD	P	AD	Yu et al., 2016
5	*RHO*	c.403C > T	p.Arg135Trp	EX2/CDS2	Het	chr3:129530917:C > T	Missense	.	DC	PD	P	AD	Yu et al., 2016
6	*RP2*	c.348_349insT	p.Phe117Phefs7	EX2/CDS2	Hemi	chrX:46853721	Frameshift	.	.	.	LP	XL	This study
	*RP1*	c.1419_1420delTG	p.Thr473Thrfs13	EX4/CDS3	Het	chr8:54625300	Frameshift	.	.	.	LP	XL	This study
7	*AGBL5*	c.1406C > G	p.Ser469Ter	EX8/CDS7	Het	chr2:27056663	Nonsense	.	.	PD	LP	AR	This study
	*AGBL5*	c.1498C > T	p.Arg500Cys	EX8/CDS7	Het	chr2:27056755	Missense	Dam	.	PD	VUS	AR	This study
8	*BEST1*	c.584C > T	p.Ala195Val	EX4/CDS3	Het	chr11:61956946:C > T	Missense	Dam		PD	P	AD	Gao, Qi, et al., 2019
9	*RP9*	c.511_512delGA	p.Glu171ArgfsX2	EX6/CDS6	Het	chr7:33135000.0.33135001	Frameshift	.	.	.	LP	AD	This study
	*CYP4V2*	c.283G > A	p.Gly95Arg	EX2/CDS2	Het	chr4:187115722	Missense	.	.	.	P	AR	Aodon et al, 2017
10	*EYS*	c.8012T > A	p.Leu2671Ter	EX41/CDS38	Het	chr6:63762520:A > T	Nonsense	.	.	PD	P	AR	Sengillo et al., 2018
	*EYS*	c.6416G > A	p.Cys2139Tyr	EX31/CDS28	Het	chr6:64230600:C > T	Missense	.	.	Pol	P	AR	Chen et al., 2015
11	*FLVCR1*	c.719delC	p.Thr240ThrfsX20	EX1/CDS1	Het	chr1:213032513	Frameshift	.	.	.	LP	AR	This study
	*RIMS1*	c.3136delA	p.Lys1046LysfsX32	EX20/CDS20	Het	chr6:72974697	Frameshift	.	.	.	LP	AR	This study
	*USH2A*	c.2802T > G	p.Cys934Trp	EX13/CDS12	Het	chr1:216419934	Missense	Dam	.	PD	P	AR	Lenassi et al., 2015
	*USH2A*	c.13939G > C	p.Gly4647Arg	EX64/CDS63	Het	chr1:215844508	Missense	.	.	.	VUS	AR	This study
	*USH2A*	c.10830G > C	p.Trp3610Cys	EX55/CDS54	Het	chr1:215953294	Missense	.	.	.	VUS	AR	This study
12	*MERTK*	c.225delA	p.Thr75Thrfs4	EX2/CDS2	Hom	chr2:111929282:CA > C	Frameshift	.	.	.	LP	AR	This study
	*EYS*	c.2756G > A	p.Gly919Glu	EX18/CDS15	Het	chr6:64902203:C > T	Missense	Dam	.	Pol	VUS	AR	This study
	*EYS*	c.6410G > A	p.Arg2137His	EX31/CDS28	Het	chr6:64230606:C > T	Missense	Dam	.	Pol	VUS	AR	This study
13	*RP1*	c.2886delA	p.Gly962GlyfsX3	EX4/CDS3	Het	chr8:55539328	Frameshift	.	.	.	LP	AD	This study
	*RP1*	c.4129delG	p.Asp1377ThrfsX20	EX4/CDS3	Het	chr8:55540571	Frameshift	.	.	.	LP	AD	This study
14	*RP1*	c.4168_4169insT	p.His1390Serfs6	EX4/CDS3	Het	chr8:54628050	Frameshift	.	.	.	LP	AR	This study
	*RP1*	c.4169A > G	p.His1390Arg	EX4/CDS3	Het	chr8:54628051	Missense	Dam	.	Pol	VUS	AR	This study
	*RP1*	c.4196delG	p.Cys1399Leufs5	EX4/CDS3	Het	chr8:54628077	Frameshift	.	.	.	P	AR	Jing et al., 2014
	*RP1*	c.6353G > A	p.Ser2118Asn	EX4/CDS3	Het	chr8:54630235	Missense	Dam	.	Pol	P	AR	Jing et al., 2014
15	*USH2A*	c.14285A > G	p.Asn4762Ser	EX65/CDS64	Hom	chr1:215650650:T > C	Missense	Dam	.	PD	P	AR	Xu et al., 2014
16	*USH2A*	c.8641_8642insTATT	p.Ser2881Tyrfs9	EX43/CDS42	Het	chr1:215877797	Frameshift	.	.	.	LP	AR	This study
	*USH2A*	c.13465G > A	p.Gly4489Ser	EX63/CDS62	Het	chr1:215674446	Missense	Tol	.	PD	VUS	AR	This study
17	*USH2A*	c.10601A > G	p.Tyr3534Cys	EX54/CDS53	Het	chr1:215782181:T > C	Missense	Dam	.	PD	VUS	AR	This study
	*USH2A*	c.8559‐2A > G	_	Intron42	Het	chr1:215877882:T > C	SpliceSite	.	.	PD	P	AR	Lulin et al., 2018
18	*USH2A*	c.7075_7076delTT	p.Leu2359Asnfs*17	EX37/CDS36	Het	chr1:215965360:TAA > T	Frameshift	.	.	.	LP	AR	This study
	*USH2A*	c.2802T > G	p.Cys934Trp	EX13/CDS12	Het	chr1:216246592:A > C	Missense	.	.	PD	P	AR	Lenassi et al., 2015
19	*USH2A*	c.4021G > C	p.Ala1341Pro	EX18/CDS17	Het	chr1:216198375:C > G	Missense	Dam	.	PD	VUS	AR	This study
	*USH2A*	c.8559‐2A > G	_	Intron42	Het	chr1:215877882:T > C	SpliceSite	.	.	PD	P	AR	Lulin et al., 2018
20	*USH2A*	c.99_100insT	p.Ser33Serfs42	EX2/CDS1	Het	chr1:216422237	Frameshift	.	.	.	P	AR	Dai, Zhang, Zhao, Deng, & Li, 2008
	*USH2A*	c.8254G > A	p.Gly2752Arg	EX42/CDS41	Het	chr1:215879068	Missense	Dam	.	PD	P	AR	Perez‐Carro et al., 2016
21	*USH2A*	c.9469C > T	p.Gln3157Ter	EX48/CDS47	Het	chr1:215990440	Nonsense	.	.	.	P	AR	Huang et al., 2013
	*USH2A*	c.11156G > A	p.Arg3719His	EX57/CDS56	Het	chr1:215933077	Missense	.	.	.	LP	AR	Lulin et al., 2018
22	*USH2A*	c.8232G > C	p.Trp2744Cys	EX42/CDS41	Het	chr1:215879090	Missense	Dam	.	PD	P	AR	Sodi, Mariottini, Passerini, Murro, & Torricelli, 2014
	*USH2A*	c.2802T > G	p.Cys934Trp	EX13/CDS12	Het	chr1:216246592	Missense	.	.	PD	P	AR	Lenassi et al., 2015
23	*RPGR*	c.905G > A	p.Cys302Tyr	EX8/CDS8	Hemi	chrX:38304664:C > T	Missense	.	DC	PD	LP	XL	This study

Abbreviations: AD, autosomal dominant; AR, autosomal recessive; Ben, benign; Dam, damaging; DC, disease causing; Del, deleterious; LP, likely pathogenic; Neu, neutral; P, pathogenic; PD, probably damaging; Pol, polymorphism; Tol, tolerated; VUS, uncertain clinical significance; XL, X‐linked inheritance.

### 3.2 Genetics analysis

A total of 45 variants of retinitis pigmentosa (RP)‐causative genes were identified in the total cohort, including 22 novel variants and 23 known variants. Overall, there were 22 pathogenic variants, 14 likely pathogenic variants, and nine uncertain clinical significance variants, the overall variation detection rate was 80% (36/45). The majority of likely pathogenic variants (*n* = 13) and all uncertain‐significance (*n* = 9) were novel variants. Three of the most common known pathogenic variations in two genes (*USH2A* and *RHO*) were detected among seven probands, the three commonest variants were *USH2A* c.2802T > G p.Cys934Trp (13%, 3/23), c.8559‐2A > G (8.7%, 2/23), and *RHO* c.403C > T c.403C > T (8.7%, 2/23), and all of these were known pathogenic variations.

### 3.3 Genotype‐phenotype correlations

The proband of the family seven detected a compound heterozygous novel mutation in the *AGBL5* gene c.1406C > G (p.Ser469Ter) and c.1498C > T (p.Arg500Cys). After Sanger verification, the patient's father carried the c.1406C > G missense heterozygous mutation with *AGBL5* gene, and the patient's mother carried a c.1498C > T missense heterozygous mutation with *AGBL5* gene, but the patient's parental eye examination was normal (Figure S1). Through the co‐segregation verification and clinical phenotypic analysis, we consider *AGBL5* gene mutations as candidate pathogenic variants in this family of retinitis pigmentosa. The proband had symptoms of night blindness from the age of 12, but the visual acuity was not affected, and the best corrected visual acuity remained at 0.6/0.8. Clinical fundus photography and fundus puzzles show that except for the posterior macular area, a medium amount of bright yellow granule‐like crystals were deposited in the equatorial region and the peripheral retina, with only a small amount of scattered pigmentation was observed, and the retina showed a mottled appearance. Autofluorescence photography showed that the autofluorescence of retinal pigment cells was confined to the central region of the posterior pole, and the peripheral fluorescence was significantly attenuated. This performance was highly consistent with the choroidal atrophy and visual field. Binocular visual field examination showed that the visual field was tubular and the peripheral visual field was completely lost; the flash ERG detection indicated that the Rod–Cone response of both eyes was quenched; The corneal topographic examination suggested that the corneas of both eyes were highly retrograde astigmatism, and the astigmatism was −2.5D (right eye) and −2.1D (left eye), respectively (Figure S2).

In the two families of retinitis pigmentosa in the 13th and 14th, three pairs of compound heterozygous novel mutations of the *RP1* gene were detected, and six variants were all in exon 4. Among them, the proband of the 13th family detected a compound heterozygous novel frameshift mutations in the *RP1* gene c.2886delA (p.Gly962GlyfsX3), and c.4129delG (p.Asp1377ThrfsX20). Combined with clinical phenotypic analysis, we confirmed that the compound heterozygous mutation was a causative mutation of binocular retinitis pigmentosa. At the age of 8 years, the proband began to experience night blindness. At the age of 20, his vision decreased. At the age of 30, he developed a macular hole. The fundus photography of the eyes showed a small amount of osteoblast‐like pigmentation in the retina, involving the macula. The blood vessels and choroids are slightly atrophied, and autofluorescence indicates that the retinal pigment background fade, and the visual field examination is irregular (Figure S3). The proband of the 14th family detected two pair of compound heterozygous mutations in the *RP1* gene: c.4168_4169insT (p.His1390Serfs6), c.4169A > G (p.His1390Arg), c.4196delG (p.Cys1399Leufs5) and c.6353G > A (p.Ser2118Asn). Three of these variants occurred in a gene mutation region ranging from 4,168 to 4,196 in a total of 28 base sequences. By Sanger verification, the c.4168_4169insT and c.4169A > G mutations were detected in the patient's father, while the c.4196delG and c.6353G > A mutations were detected in the patient's mother, but the patient's clinical examination was normal. The patient developed night blindness within 1 year of age. At 12 years old, his vision decreased, with posterior subcapsular cataract and full color blindness. Except for the posterior macular area, the remaining retina had a blue–gray color and did not show obvious osteocyte‐like pigmentation. Fluorescein fundus angiography suggested that the fluorescent background of retinal pigmentation in the fundus was extremely low or absent, showing a mottled appearance. OCT showed that the fovea of the eyes was extremely atrophied, and the thickness was only 37 μm for the right eye and 31 μm for the left eye (Figure S4).

## 4 DISCUSSION

Of the 23 retinitis pigmentosa families included in the study, 17 probands had clinical manifestations of concurrent lens opacities, and the posterior subcapsular opacities was predominant (nine cases, accounting for 39.13%), suggesting that posterior subcapsular cataracts is the commonest forms of retinitis pigmentosa combined with complication cataract. At the same time, the clinical manifestations of probands with retinitis pigmentosa combined with complication choroidal atrophy were also inconsistent, with peripheral choroidal atrophy as the commonest form (12 cases, accounting for 57.14%). Of these probands with complication peripheral choroidal atrophy, there were eight probands (8/12, 66.7%) caused by the pathogenic variation of *USH2A* gene. However, there were no significant genotypic differences in probands with concurrent lens opacities. In terms of best corrected visual acuity, peripheral choroidal atrophy has better best corrected visual acuity than patients with posterior choroidal atrophy (the proportion of eyes with visual acuity greater than 0.05 was 79.17% and 12.50%, respectively). It indicated that the best corrected visual acuity may be related to the degree of choroidal atrophy and the degree of pigment epithelium and cone atrophy. Among the probands with mutations detected, compound heterozygous forms was detected in 13 (56.5%, 13/23) probands, and digenic inheritance forms was detected in five (21.7%, 5/23) probands. It suggested that the compound heterozygous forms and digenic inheritance forms are one of the most striking features of the genetic pathogenesis of retinitis pigmentosa (Audo et al., 2012; Corton et al., 2013; Daiger, Sullivan, & Bowne, 2013; Veltel & Wittinghofer, 2009).

The *RP1* gene is one of the most common pathogenic genes of retinitis pigmentosa, which encodes oxyregulin 1, involved in the development of photoreceptors and the transport of proteins or the maintenance of cilia between the inner and outer segments, as well as the formation of tissue structures in the outer segments of photoreceptors, and the regulation of photoreceptor microtubules, etc., (Astuti et al., 2016; Liu, Zhou, Daiger, Farber, & Pierce, 2002; Pierce et al., 1999) it's main genetic methods include adRP and arRP (Bowne, 1999; Khaliq, 2005). In the present study, mutations associated with the *RP1* gene were detected in probands No.13 and No.14. The difference is that the c.2886delA and c.4129delG mutations of the *RP1* gene detected in 13th proband were frameshift mutations, causing a change in the reading frame, resulting in a change in the amino acid sequence of the encoded protein. For the 14th patient, there were four pathogenic mutations in the *RP1* gene, c.4168_4169insT and c.4196delG mutations were insertion and deletion frameshift mutations, and c.4169A > G and c.6353G > A mutations were missense mutations. The patient's four heterozygous mutations in the *RP1* gene are located at different loci, eventually forming two pairs of compound heterozygous mutations. The effect of two pairs of compound heterozygous mutants may be more severe than that of a pair of compound heterozygous mutations. Corresponding to clinical manifestations, the degree of fundus lesions in proband No. 14 was also much severe than that in patients with No. 13. The age of onset of night blindness in proband No. 14 was less than 1‐year‐old, with full color blindness, severe macular atrophy, disappearance of the outer nuclear layer and cone membrane disc structure of the fovea, and autofluorescence suggesting that the fluorescence of the whole pigment epithelium disappeared, especially obviously of the arterial vascular atrophy. However, the 13th patient began to develop night blindness at the age of 8 years, without color blindness, mild atrophy of blood vessels and choroids, and autofluorescence suggesting that the retinal pigment background fades.

In this study, Proband No. seven is a crystalline retinitis pigmentosa caused by mutation of *AGBL5* gene, also known as retinitis pigmentosa 75. Its common genetic pattern is autosomal recessive inheritance. *AGBL5* gene encodes protein ATP/GTP binding protein‐like five, which is involved in the regulation of microtubules outside photoreceptors (Branham et al., 2016; Kastner et al., 2015; Patel et al., 2016). The protein is a member of the cytoplasmic carboxypeptidase (CCP) family, which also includes *AGTPBP1* (Nna‐1, CCP1, MIM#606830), *AGBL1* (CCP4, MIM#615496), and *AGBL4* (CCP6, MIM#616476). The main function of these proteins is to remove long‐chain glutamate chains from tubulin, whereas the *AGBL5* protein functions in reverse. It mainly localizes the glutamate branching point on tubulin, thereby prolonging the glutamic acid chain (Rogowski et al., 2010). There are few reports of retinitis pigmentosa caused by mutation of *AGBL5* gene, and only a few reports indicate that homozygous nonsense mutation of *AGBL5* gene can cause non‐syndromic retinitis pigmentosa (Kastner et al., 2015). In turn, it affects the function of tubulin, which eventually leads to atrophy of the optic nerve cell layer and internal and external plexiform layers, causing retinal degenerative diseases. The *AGBL5* gene mutation found in this study belongs to a compound heterozygous mutation consisting of p.Ser469Ter (c.1406C > G) and p.Arg500Cys (c.1498C > T), all of which were first discovered novel variations. The proband showed blurred vision at the age of eight and decreased in visual acuity at 12 years of age with posterior subcapsular cataract (PSC). Through the co‐segregation and Sanger verification, it was found that each parent carries a mutation in the gene locus, but the parents have no abnormal clinical manifestations. The crystallized retinitis pigmentosa (BCD) of the probands is different from the BCD manifestation caused by the typical *CYP4V2* gene mutation. Crystalline‐like substances are mainly concentrated in the equatorial region and the peripheral retina, but there is no significant change in the posterior polar region. We consider that the tubulin encoded by the *AGBL5* gene only affects the cilia activity and metabolism of rod cells, but has less effect on cone cells (Branham et al., 2016; Patel et al., 2016).

In summary, this study provides novel mutations and clinical phenotypes of retinitis pigmentosa, and the result suggest that choroidal atrophy can be used as one of the indicators for best correcting visual acuity and retinitis pigmentosa. Posterior subcapsular opacities is the most common form of secondary cataracts, and peripheral choroidal atrophy is the most common form of secondary choroidal atrophy. At the same time, more than half of the probands with retinitis pigmentosa are caused by compound heterozygous forms, and about one‐fifth of the probands are caused by digenic inheritance forms. Our finding not only extend the existing genotype spectrum, but also provide an effective reference for the design of panel‐based genetic diagnostic testing, genetic counseling, and future gene therapy in northeast Chinese patients with retinitis pigmentosa, and have a deeper understanding of the relationship between clinical manifestations and genotypes.

## CONFLICT OF INTEREST

The authors declare that the research was conducted in the absence of any commercial or financial relationships that could be construed as a potential conflict of interest.

## AUTHOR CONTRIBUTIONS

WH, FC, WL, and YS conceived and designed this study. YS, Z‐SW, LX, and WH recruited patients, performed clinical examinations and interpretation. WL, YS, BX, and J‐YB collected the clinical samples and clinical data. J‐KL, WY, L‐SW, Z‐WW, and W.L analyzed the sequencing data. WL and YS wrote and revised the manuscript. All authors read and approved the final manuscript.

## Data Availability

The data that support the findings of this study have been deposited in the CNSA (https://db.cngb.org/cnsa/) of CNGBdb with accession code CNP CNP0000503.
