# Supplementary figures and images for: Genetic and clinical findings of panel‐based targeted exome sequencing in a northeast Chinese cohort with retinitis pigmentosa
Source: Mol Genet Genomic Med. 2020 Feb 26;8(4):e1184. doi: 10.1002/mgg3.1184 (PMC7196472; doi:10.1002/mgg3.1184)

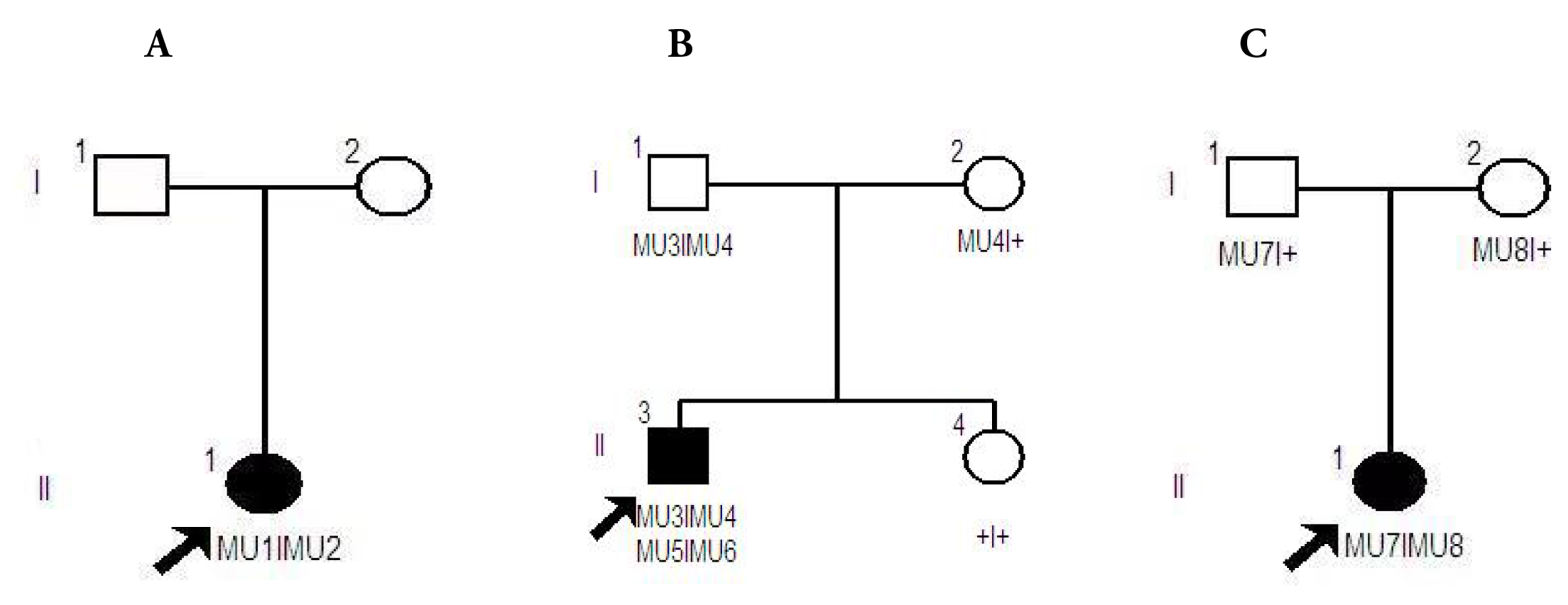

Supplement: Supplementary file 2 [file MGG3-8-e1184-s002.png]

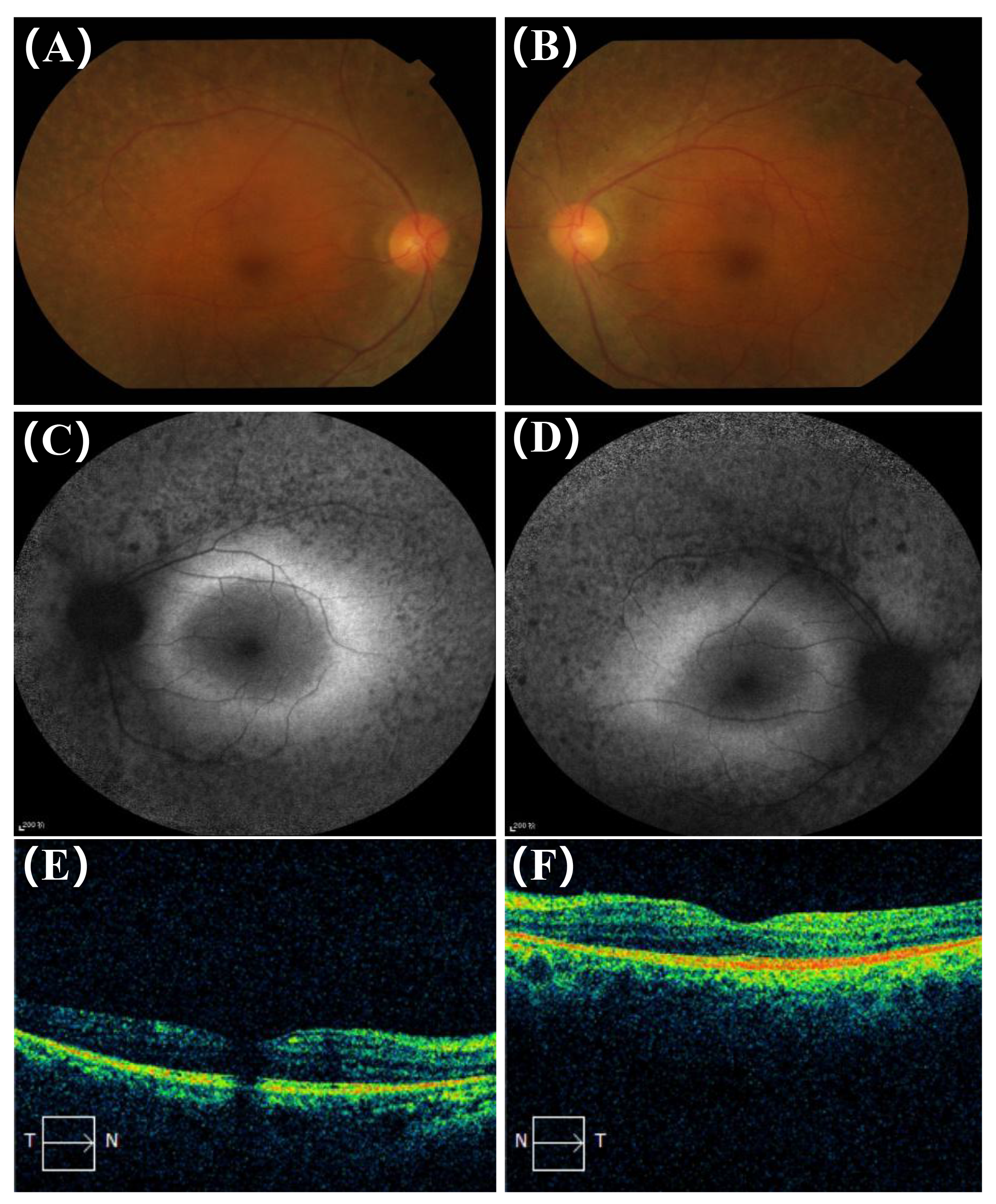

Supplement: Supplementary file 3 [file MGG3-8-e1184-s003.png]

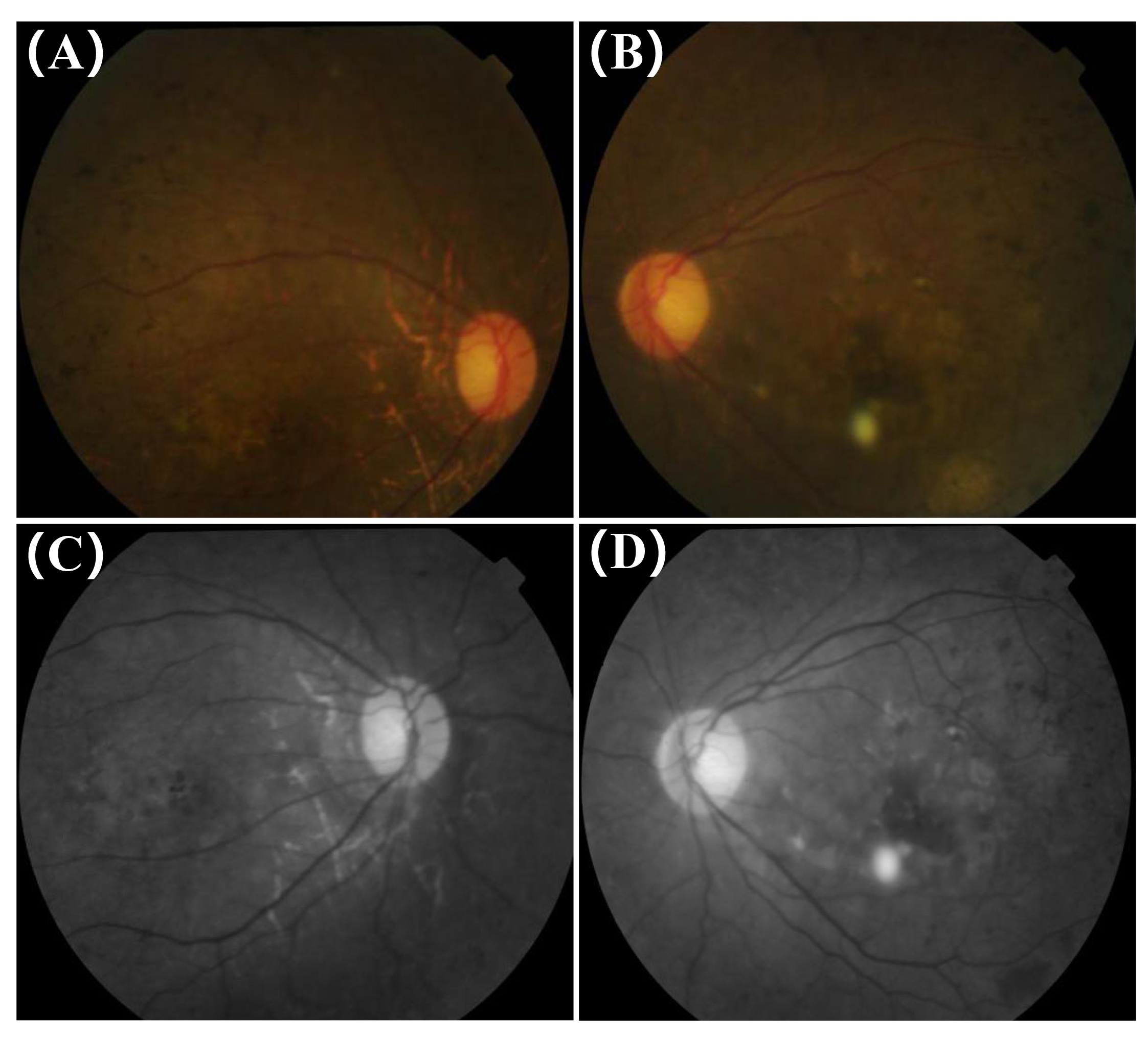

Supplement: Supplementary file 4 [file MGG3-8-e1184-s004.png]

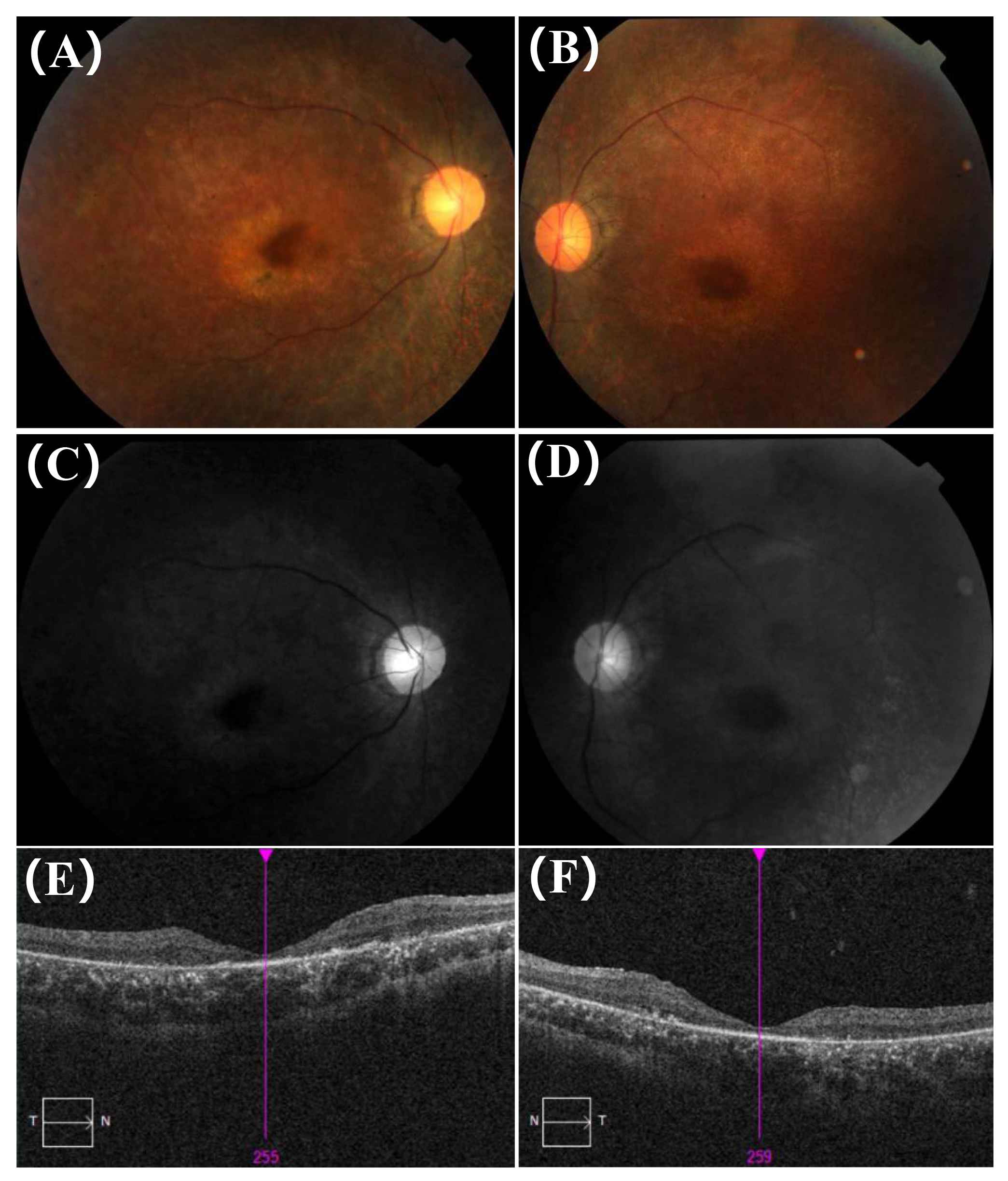

Supplement: Supplementary file 5 [file MGG3-8-e1184-s005.png]
